# The Exopolysaccharide Produced by *Lactobacillus paracasei* IJH-SONE68 Prevents and Ameliorates Inflammatory Responses in DSS–Induced Ulcerative Colitis

**DOI:** 10.3390/microorganisms9112243

**Published:** 2021-10-28

**Authors:** Masafumi Noda, Narandalai Danshiitsoodol, Keishi Kanno, Tomoyuki Uchida, Masanori Sugiyama

**Affiliations:** 1 Department of Probiotic Science for Preventive Medicine, Graduate School of Biomedical and Health Sciences, Hiroshima University, Hiroshima 734-8551, Japan; bel@hiroshima-u.ac.jp (M.N.); naraa@hiroshima-u.ac.jp (N.D.); 2 Department of General Internal Medicine, Hiroshima University Hospital, Kasumi 1-2-3, Minami-ku, Hiroshima 734-8551, Japan; kkanno@hiroshima-u.ac.jp; 3 Department of Clinical Pharmaceutical and Therapeutics, Hiroshima University, Kasumi 1-2-3, Minami-ku, Hiroshima 734-8551, Japan; t.uchida@asahi-techneion.co.jp; 4 Sone Farm Co., Ltd., Shinjuku, Shinjuku-ku, Tokyo 160-0022, Japan

**Keywords:** inflammatory bowel disease (IBD), ulcerative colitis (UC), lactic acid bacteria (LAB), *Lactobacillus paracasei*, exopolysaccharide (EPS)

## Abstract

Inflammatory bowel disease (IBD) is an autoimmune disease characterized by chronic inflammation of the gastrointestinal tract. IBD includes Crohn’s disease (CD) and ulcerative colitis (UC). CD can occur in any part of the gastrointestinal tract, whereas UC mainly occurs in the colon and rectum. We previously demonstrated that a novel exopolysaccharide (EPS) produced by a plant-derived bacterium, *Lactobacillus paracasei* IJH-SONE68, prevents and improves the inflammation in contact dermatitis model mice via oral administration. To evaluate the preventive effect of the EPS against other inflammatory diseases, in the present study, we employed dextran sulfate sodium (DSS)-induced UC model mice. The stool consistency, hematochezia, and colonic atrophy of the mice were improved by the orally administered EPS. We also evaluated the cytokine transcription. Overexpression of the mouse macrophage inflammatory protein 2 mRNA in the colon as a functional homolog of human interleukin-8 was decreased by the orally administered EPS. However, the expression of interleukin-10, which is known as an anti-inflammatory cytokine, was stimulated in the EPS-administrated group. Based on these results, we conclude that the IJH-SONE68-derived EPS is a promising lead material for the development of drugs useful in treating inflammatory diseases such as UC.

## 1. Introduction

The number of individuals worldwide who suffer from inflammatory bowel disease (IBD) is increasing. Historically, IBD was most prevalent in North Americans and Europeans. However, the recent shift in dietary and lifestyle patterns in newly industrialized countries, such as those in Asia, Africa, South America, and Eastern Europe, has caused a significant rise in the incidence of IBD [1]. In the latest report, there were 6.8 million cases of IBD globally in 2017 [2].

IBD patients may present with symptoms such as chronic diarrhea, rectal bleeding, abdominal pain, and weight loss. IBD is defined as an autoimmune disease characterized by chronic inflammation of the gastrointestinal tract caused by a dysfunction of the innate immune system [3]. The two major subtypes of IBD are Crohn’s disease (CD) and ulcerative colitis (UC), both having complex pathological spectra [3,4,5]. CD can occur in any part of the gastrointestinal tract, from the mouth to the anus. Predilection sites for the disease are the small intestine, colon, and anus. The areas of inflammation in the gastrointestinal tract are patchy and next to healthy tissue. UC usually occurs in the colon, and the damaged areas continuously spread to the rectum.

The exact causes of both diseases are unknown. However, it has been suggested that when some environmental triggers incorrectly activate the immune system in patients, their abnormal immune responses continuously attack their gastrointestinal tracts, resulting in chronic inflammation. Since CD and UC are chronic inflammatory diseases, they are not medically curable once they have developed [6]. Patients must continue medical treatment with the aim of decreasing inflammation, but the drugs show limited efficacy in remission and some significant side effects with long-term use [7]. Therefore, the development of more effective therapeutic agents and treatments as soon as possible is desired.

Lactic acid bacteria (LAB) are generally nonpathogenic Gram-positive bacteria and are known as typical probiotics, which are defined as living microorganisms that confer health benefits to human hosts [8]. We previously isolated some exopolysaccharide (EPS)-producing LAB strains. Interestingly, those EPSs have displayed a hyaluronidase-inhibitory effect [9,10,11] that has been reported to correlate with histamine-release inhibition in inflammatory reactions through immunoglobulin E (IgE)-mediated mast cell degranulation [12,13,14]. Among EPS-producing LAB strains, we found that the *Lactobacillus* (*Lb.*) *paracasei* IJH-SONE68–derived EPS prevents and improves inflammation in picryl chloride-induced contact dermatitis via oral administration in model mice [15]. We considered that the IJH-SONE68 strain-derived EPS may modulate immune responses under inflammatory conditions. Therefore, to evaluate in this study whether the EPS has a preventive effect against other inflammatory diseases other than contact dermatitis [15], we used dextran sulfate sodium-induced UC model mice to evaluate the preventive and improving effects of the IJH-SONE68-derived EPS against UC.

## 2. Materials and Methods

### 2.1. Bacterial Strains and Culture Conditions

For precultivation of *Lb. paracasei* IJH-SONE68, a de Man, Rogosa, and Sharpe (MRS) medium (Merck KGaA, Darmstadt, Germany) was used. The IJH-SONE68 strain has been deposited in the National Institute of Technology and Evaluation (NITE) Patent Microorganisms Depositary (NPMD) in Japan as NITE P-02242. Brain heart infusion (BHI) broth was used as the culture medium for *Salmonella enterica* serovar Typhimurium (*S.* Typhimurium) and *Campylobacter* (*C.*) *jejuni*.

### 2.2. Purification of EPSs

Purification of the EPSs produced from the IJH-SONE68 strain was performed according to previous reports [9,15]. Briefly, the IJH-SONE68 strain was inoculated into a fresh modified semi-defined medium (SDM) [16,17] supplemented with 0.2% (*v*/*v*) vitamin mixture and 0.1% (*v*/*v*) trace element solution instead of yeast nitrogen bases. After 2 days of stationary cultivation, the cultured broth was boiled for 30 min, and then a 100% (*w*/*v*) trichloroacetic acid (TCA) solution was added to the culture broth at a final concentration of 4% (*v*/*v*). The cells were removed by centrifugation, and the obtained culture supernatant was added to the same volume of acetone in order to precipitate crude EPSs. After centrifugation, the crude EPSs were dissolved into a 50 mM Tris-HCl buffer (pH 8.0) and treated with nuclease and protease. The EPSs were dialyzed against sterile distilled water using Amicon Ultra (MWCO = 10 kDa, Merck Millipore, Ltd., Carrigtwohill, Co. Cork, Ireland) to obtain a purified EPS mixture, and the sample was lyophilized and stored at 4 °C until the IL-8 release inhibition assay was used.

The neutral and acidic EPSs were further separated via column chromatography using an anion exchange resin (TOYOPEARL DEAE-650M, Tosoh Bioscience, Tokyo, Japan) as described previously [10]. After dialysis against sterile distilled water using Amicon Ultra (MWCO = 10 kDa), each EPS sample was lyophilized and stored at 4 °C until use.

### 2.3. IL-8 Release Inhibition Assay

The assay using Caco-2 cells was performed according to previous reports [18,19]. Briefly, Caco-2 cells were precultured in Dulbecco’s modified Eagle’s medium (DMEM) (low glucose, FUJIFILM Wako Pure Chemical Co., Ltd., Osaka, Japan) containing 10% (*v*/*v*) fetal bovine serum (FBS), 2 mM L-glutamine, 100 U/mL penicillin, and 100 μg/mL streptomycin in an atmosphere of 5% CO_2_ at 37 °C. The cultured Caco-2 cells were resuspended into the same fresh medium, and a total of 2.0 × 10^5^ cells were seeded in a 24-well cell-culture plate and cultured to 70% confluence under the same condition. Before starting the experiments, the confluent monolayers were washed and starved in serum-free DMEM. The cells were preincubated for 30 min with a 5% (*v*/*v*) purified EPS mixture (1 mg/mL) and then stimulated for up to 2 h with a freshly prepared *S. Typhimurium* or *C. jejuni* suspension at a multiplicity of infection of 10. The bacterial infection was terminated with 100 µg/mL gentamicin, and the cell monolayers were further incubated for 24 h. The cell-free supernatants were collected, and concentrations of the released IL-8 were determined via enzyme-linked immune sorbent assay (ELISA) (BioLegend, Inc., San Diego, CA, USA) according to the manufacturer’s instruction. At least four replicates of all assays were performed. The culture without bacterial stimulation was used as a negative control (NC), and one treated with an *S. Typhimurium* or *C. jejuni* suspension at a multiplicity of infection of 10 was used as a positive control (PC).

### 2.4. Animals and Rearing Conditions

Seven-week-old male-specific-pathogen-free (SPF) C57BL/6J Jms Slc mice were purchased from Japan SLC, Inc. (Shizuoka, Japan). The mice were weighed prior to experimentation and then divided into experimental groups of five mice each and housed in a plastic cage. All mice were maintained with free access to water and a standard rodent-chow diet (MF diet, Oriental Yeast Co., Ltd., Tokyo, Japan) under conditions of 20–26 °C, 40–60% humidity, and a 12 h light/12 h dark cycle. Each mouse was distinguished by different-color markings on their tails using Animal Marker felt pens (Fuchigami Kikai Co., Ltd., Kyoto, Japan). After the experimental period, the mice were euthanized by the inhalation of anesthesia with isoflurane. The animal experimental protocol was approved by the committee of the Research Facilities for Laboratory Animal Science of Hiroshima University (approval number A18-2, approval on 3 April 2018), and the animal experiment in the present study was conducted in accordance with the Guidelines for the Care and Use of Laboratory Animals of Hiroshima University after approval of the protocol.

### 2.5. Preparation and Evaluation of the DSS-Induced UC Model in Mice

The 20 mice were divided into four experimental groups (five mice each) as follows: a group without UC induction (negative control group, NC); a group with UC induction but without treatment (positive control group, PC); a group with UC induction and neutral-EPS (N-EPS) administration; and a group with UC induction and acidic-EPS (A-EPS) administration.

After 7 days of pre-administration of the EPS solution, DSS was added to the mice’s drinking water at a final concentration of 3% (*w*/*v*) to induce UC, except for the NC group. During the other two-week UC-induction period, the body weight and the stool consistency, and hematochezia scores of all mice were recorded. The totals of both scores were evaluated as a disease activity index (Table 1).

After the experimental period, the mice were euthanized by the inhalation of anesthesia with isoflurane. The length of colon collected from each mouse was measured, and the middle part was cut into about 5 mm pieces and stored for use in further analyses.

### 2.6. Myeloperoxidase (MPO) Activity Assay

Each PBS-washed colon piece (10–25 mg) was weighed and transferred into a 1.5 mL microcentrifuge tube with 100 mg of glass beads (acid-washed ≤106 μm, Sigma-Aldrich Co., LLC, St. Louis, MO, USA). Each colon sample was homogenized with a pestle in a microtube, and then a 50 mM potassium phosphate buffer (pH 6.0) containing 0.5% (*w*/*v*) hexadecyltrimethylammonium bromide (HTAB) was added at a volume of 40 μL/mg of tissue. After gentle mixing, the glass beads and cell debris were removed from the homogenized sample by centrifugation at 13,000× *g* for 10 min at room temperature (RT). The obtained supernatant was transferred into a fresh tube and stored at −80 °C until use as an enzyme solution.

The MPO activity was measured in accordance with the previously reported protocol [20]. The reaction buffer was prepared by adding *o*-dianisidine dihydrochloride and H_2_O_2_ to a 5 mM potassium phosphate buffer (pH 6.0) at concentrations of 0.53 mM and 44 μM, respectively. After mixing 7 μL of the enzyme solution and 200 μL of the reaction buffer in a 96-well plate, absorbance was measured at 450 nm (*A*_450_) using a spectrophotometer (iMark Microplate reader, Bio-Rad Laboratories, Inc., Hercules, CA, USA) at 3 min intervals. The MPO activity was calculated in units per mg of tissue. One unit of MPO is defined as the enzyme amount that degrades 1 μmol/min of H_2_O_2_, determined as (Δ*A*_450_/min) × (1.13 × 10^−2^) [20].

### 2.7. Preparation, Manipulation, and qRT-PCR Analysis of RNA

Total RNA was extracted and purified from each colon tissue using a NucleoSpin RNA II kit (Macherey-Nagel GmbH & Co. KG, Düren, Germany) in accordance with the manufacturer’s instructions. The RNA was converted to cDNA using the ReverTra Ace qPCR RT master mix with gDNA remover (Toyobo, Osaka, Japan) in accordance with the manufacturer’s instruction manual. The qRT-PCR was conducted on the PikoReal Real-Time PCR System (Thermo Fisher Scientific, Waltham, MA, USA), and the reaction was performed using the KAPA SYBR Fast qPCR Kit (Kapa Biosystems, Wilmington, MA, USA). The PCR was performed under the following conditions: an initial denaturation for 30 s at 95 °C, followed by 40 cycles of 5 s denaturing at 95 °C and 30 s at 60 °C. The relative transcriptional levels of the target genes were normalized to that of the reference gene (β-actin) using the ΔΔC_T_ method. The primer sets in this experiment are listed in Table 2.

### 2.8. Statistical Analyses

Statistical analyses conducted in the present study were performed using SPSS 17.0 software (IBM Corporation, New York, NY, USA). Multiple comparisons of parameters were assessed using the Tukey–Kramer test [21].

### 2.9. Histopathological Analysis

Mouse colons obtained after sacrifice were fixed with a 4% (*w*/*v*) formaldehyde solution and then embedded in a paraffin block. The histopathological tissue sections were cut from the block and placed on glass slides. The sections were stained with hematoxylin and eosin (HE). Tissue sections were monitored and recorded using an OLYMPUS IX71 research inverted system microscope (Olympus Corporation, Tokyo, Japan) with controlling and image-capturing software. The average crypt depth was determined from at least 50 independent measures per mouse tissue.

## 3. Results

### 3.1. Effect of the IJH-SONE68-Derived EPS on the Release of IL-8 from Caco-2 Cells

An ELISA was preliminarily used to evaluate any effect on IL-8 induction in the in vitro model. In the assay, a 2-h stimulation of Caco-2 cells with S. Typhimurium or C. jejuni sufficiently induced IL-8 production, as compared with no stimulation. On the other hand, among the tested samples, the addition of the EPS mixture from the IJH-SONE68 strain significantly suppressed the pathogen-induced IL-8 expression (Supplementary Figure S1) without any inhibition of the viability of the Caco-2 cells.

### 3.2. Preventive Effect of the IJH-SONE68-Derived EPS on DSS-Induced UC Model Mice

#### 3.2.1. Disease Activity Index

**Figure 1:**
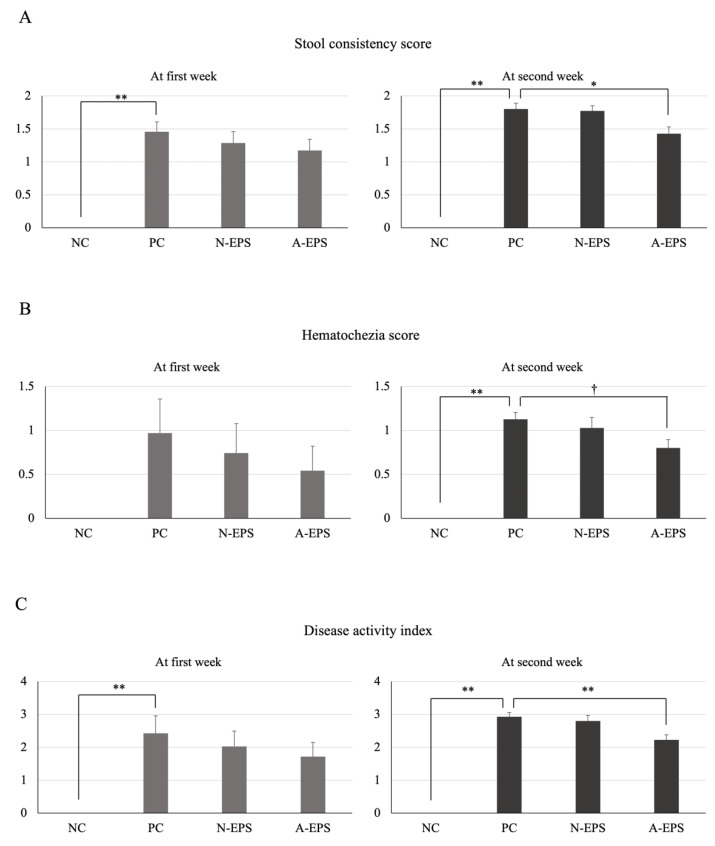
Effects of IJH-SONE68-derived EPSs in DSS-induced UC model mice on the changes in stool consistency score, hematochezia score, and disease activity index, which is defined as the total of the two scores (stool consistency and hematochezia). The right and left panels display changes in stool consistency score (**A**), hematochezia score (**B**), and disease activity index (**C**) at the first and the second week, respectively. The scores are indicated as weekly averaged ones for each group (mean ± S.E., *n* = 7). The scoring criteria for both are indicated in Table 1. Abbreviations used in the figure: NC, negative control group (without UC induction); PC, positive control group (without treatment); N-EPS, UC induction with N-EPS treatment; A-EPS, UC induction with A-EPS treatment. The statistical analyses were performed using the Tukey–Kramer multiple comparison test (^†^ *p* < 0.1, * *p* < 0.05, ** *p* < 0.01, vs. PC).

To evaluate the preventive and improving effects of EPSs on UC, DSS-induced UC model mice [22,23] were used. Figure 1 shows the changes in stool consistency and hematochezia scores in each group. Both scores increased for the DSS-induced UC model mice. EPS administration repressed the scores slightly, but not significantly, during the first week of the experimental period. However, the stool consistency was significantly (*p* < 0.05) improved during the second week with oral administration of the A-EPS, whereas the hematochezia score improvement was not significant but did demonstrate a trend (*p* = 0.057). Moreover, the disease activity index (DAI), defined as the total of the stool consistency and hematochezia scores (Table 1), of the A-EPS administration also improved significantly (*p* < 0.01) during the same period (Figure 1C).

#### 3.2.2. Colon Length

**Figure 2:**
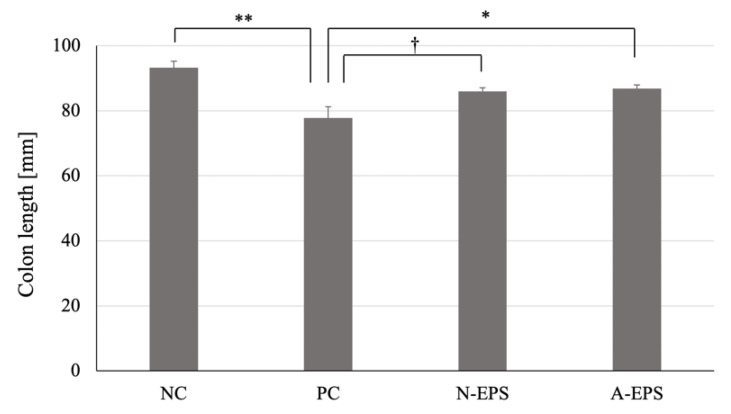
Effects of IJH-SONE68-derived EPSs in DSS-induced UC model mice on colon length after 14 days of treatment. Abbreviations used in the figure: NC, negative control group (without UC induction); PC, positive control group (without treatment); N-EPS, UC induction with N-EPS treatment; A-EPS, UC induction with A-EPS treatment. The statistical analyses were performed using the Tukey–Kramer multiple comparison test (^†^ *p* < 0.1, * *p* < 0.05, ** *p* < 0.01, vs. PC). Data are indicated by mean ± S.E. (*n* = 4–5).

Figure 2 shows the average colon length of the mice in each group. As compared with the normal group, the colon length of the DSS-treated mice was significantly (*p* < 0.01) shortened. Conversely, the administration of N-EPS or A-EPS resulted in the prevention of DSS-induced colon reduction with statistical trends (*p* = 0.052) or significance (*p* < 0.05), respectively.

#### 3.2.3. MPO Activity

**Figure 3:**
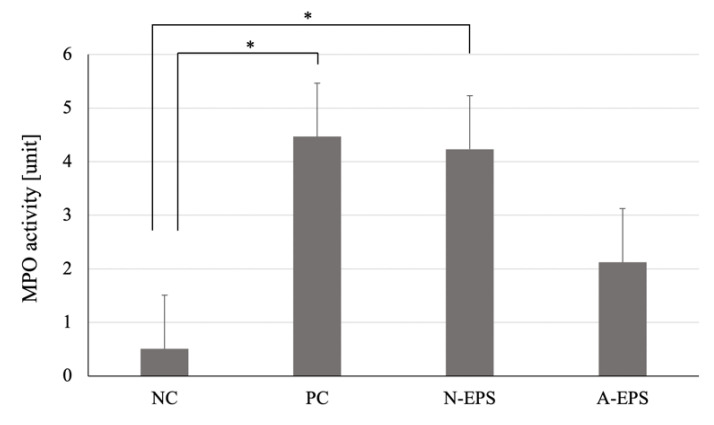
Effects of IJH-SONE68-derived EPSs in DSS-induced UC model mice on the MPO activity of colon tissue after 14 days of treatment. Abbreviations used in the figure: NC, negative control group (without UC induction); PC, positive control group (without treatment); N-EPS, UC induction with N-EPS treatment; A-EPS, UC induction with A-EPS treatment. The statistical analyses were performed using the Tukey–Kramer multiple comparisons test (* *p* < 0.05). Data are indicated by mean ± S.E. (*n* = 4–5).

MPO activity has been reported to be used as a surrogate marker of inflammation [24]. After the extraction of proteins from the tissue of each colon, the MPO activity was measured using a colorimetric assay. Figure 3 shows that the MPO activity was significantly (*p* < 0.05) increased in the DSS-treated positive control group. At the same time, in the A-EPS-administration group, but not in the N-EPS-administration group, MPO activity was repressed by about 50% with no statistical significance, which manifests prevention against DSS-induced chronic inflammation.

### 3.3. Difference in the Expression Level of Inflammatory-Related Cytokines

**Figure 4:**
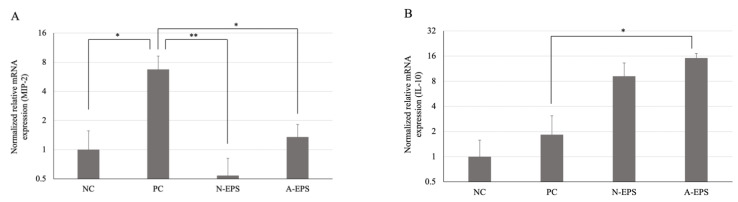
Effects of IJH-SONE68-derived EPSs in DSS-induced UC model mice on the mRNA level expressed in the colon tissue after 14 days of treatment. Each expression level of MIP-2 (**A**) and IL-10 (**B**) was normalized to that of the β-actin gene (reference gene) using the ΔΔC_T_ method. Abbreviations used in the figure: NC, negative control group (without UC induction); PC, positive control group (without treatment); N-EPS, UC induction with N-EPS treatment; A-EPS, UC induction with A-EPS treatment. The statistical analyses were performed using the Tukey–Kramer multiple comparison test (* *p* < 0.05, ** *p* < 0.01, vs. PC). Data are indicated by the mean ± S.E. (*n* = 4–5).

In the preliminary animal experiment, we have confirmed the effectiveness of the IJH-SONE68-derived EPS, especially the A-EPS, in the disease activity index, as compared with EPSs from different LAB strains (Supplementary Figure S2). The preliminarily observed results correspond well to the data obtained in the present study; therefore, to understand the mechanism of the anti-inflammation activity observed with administration of the IJH-SONE68-derived EPS, we further investigated the gene-expression levels of some inflammatory cytokines in the colon tissue, using quantitative reverse transcriptional (qRT)-PCR analysis (Figure 4). The expression level of mouse macrophage inflammatory protein 2 (MIP-2), which is a functional homolog of human IL-8, was approximately eight times higher in the PC group than in the NC group, showing that the difference was significant (*p* < 0.01). On the other hand, the administration of N- and A-EPSs resulted in the significantly low expression of MIP-2 (Figure 4A).

Figure 4B indicates the expression levels of anti-inflammatory cytokine IL-10 in each group. In contrast to the case with MIP-2, the EPS administration relatively and significantly enhanced the IL-10 levels in the N- and A-EPS groups, respectively.

The expression levels of other inflammation-related cytokines and genes, including COX-2 and iNOS, were also measured, but no significant differences were observed in the EPS groups as compared with the NC and PC groups (Supplementary Figure S3).

### 3.4. Histopathological Analysis of the Colon

**Figure 5:**
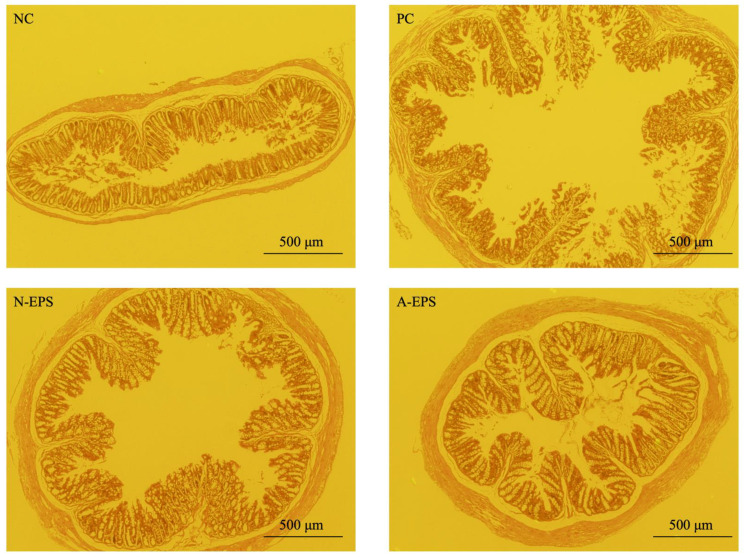
Effects of IJH-SONE68-derived EPSs on colonic tissue in DSS-induced UC model mice after 14 days of treatment. Representative colonic tissue sections were stained with hematoxylin and eosin. Abbreviations used in the figure: NC, negative control group (without UC induction); PC, positive control group (without treatment); N-EPS, UC induction with N-EPS treatment; A-EPS, UC induction with A-EPS treatment. Scale bar = 500 μm.

Pieces of mouse colon were fixed with formaldehyde and embedded in a paraffin block. The tissue sections were stained with HE and subjected to histopathological comparison (Figure 5).

**Figure 6:**
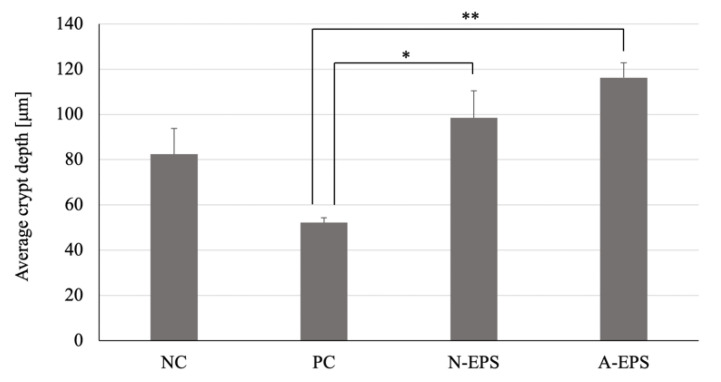
Effects of IJH-SONE68-derived EPSs in DSS-induced UC model mice on the average crypt depth after 14 days of treatment. Abbreviations used in the figure: NC, negative control group (without UC induction); PC, positive control group (without treatment); N-EPS, UC induction with N-EPS treatment; A-EPS, UC induction with A-EPS treatment. The statistical analyses were performed using the Tukey–Kramer multiple comparison test (* *p* < 0.05, ** *p* < 0.01, vs. PC). Data are indicated by the mean ± S.E. (*n* = 4–5).

The analysis showed that DSS treatment causes histopathological injuries in the colon, such as denuding of the surface epithelium and disruption of the gland structure, which are typical characteristics of DSS-derived UC (PC in Figure 5). On the other hand, although the injury is still partially observed in the EPS-administration groups, the surface of the mucosa is covered with a relatively thick epithelial layer (N-EPS and A-EPS in Figure 5). Furthermore, EPS administration seemed to protect against the decrease in crypt depth that was also caused by DSS treatment (Figure 6). When compared with the NC group, the average crypt depth of the DSS-induced PC group decreased. However, the average depths observed in EPS-administration groups were increased significantly over those of the PC group.

## 4. Discussion

Currently, due to the incurable nature of UC, the standard treatment aims to induce and maintain remission with medication. In mild to moderate UC, aminosalicylate drugs have been used as effective therapeutic agents [25,26,27]. In more severe cases, steroids, immuno-suppressive drugs, and biologics are often prescribed for patients. Several types of biologics, such as the TNF-α inhibitor infliximab or the α4β7 integrin inhibitor vedolizumab, are commonly used to treat UC patients who no longer respond to steroids [28]. Although these common agents have a high response rate in UC, some of them can cause serious side effects, such as extra-intestinal cancers, heart failure, and immune depression [29,30]. Therefore, demand for the development of safer and more effective anti-UC drugs has increased.

The UC model mice used in the present study showed a significantly higher expression level of colon MIP-2, which is a functional homolog of human IL-8, as compared to that in the negative control (Figure 4A), indicating a high correlation of the observed UC symptoms to the MIP-2 levels. On the other hand, the administration of IJH-SONE68-derived EPSs resulted in a significantly repressed MIP-2 expression level. In IBD patients, the number of infiltrated neutrophils significantly correlates with the IL-8 level in the homogenates of colonic biopsy specimens [31]. IL-8 can play a biological role through binding to IL-8 receptors A and B (also called CXCR1 and -2, respectively), which locate on the cell surface of neutrophils [32,33]. Because the IL-8–CXCR1/2 signaling axis is involved not only in inflammatory diseases, including IBD, but also in carcinogenesis, its antagonists have been used as novel therapeutic targets for gastrointestinal tract inflammatory and malignant processes in clinical trials [34].

In contrast to MIP-2, the administration of each EPS enhanced IL-10 expression, especially in the A-EPS (Figure 4A). The IL-10-induction effect of the N-EPS was lower than that of the A-EPS, and the difference may cause a weaker preventive effect on the DAI, colon length, and MPO activity in both treated groups (Figure 1, Figure 2 and Figure 3). It has been reported that the anti-inflammatory cytokine IL-10 may repress colitis inflammation in patients with UC [35]. In fact, genome-wide association studies (GWAS) of European patients have revealed that mutation in the IL-10 loci is associated with UC and CD [36,37]. The knockout of IL-10 in mice causes Th1- or Th17-type chronic inflammation and results in intestinal inflammation and colon cancer; therefore, mice have been used as one of the best animal models of IBD [38,39,40,41,42]. The intestinal macrophages from IL-10-deficient mice, which are characterized by immune dysregulation, produce large amounts of inflammatory cytokines in response to intestinal bacteria [43]. Although the exact regulatory mechanism of the EPS on cytokine expression is unknown, based on other studies of LAB-derived EPSs [44,45], including our previous report on contact dermatitis model mice [15], the IJH-SONE68-derived EPSs are expected to work through immune modulation.

Histopathological analysis (Figure 5 and Figure 6) showed that DSS treatment injured the colon and caused a decrease in crypt depth. The crypt is a tube-like gland in the colon, and pluripotent stem cells are located at the bottom of the crypt. The stem cells have been reported to reproduce themselves and renew the colonic epithelium, providing tissue homeostasis and a mucosal barrier [46,47,48]. Since mis-migration often causes intestinal disorders such as IBD, the structure of the crypt pocket is crucial for protecting stem cells from various intestinal substances [49,50]. The present study indicates that the administration of EPSs promoted the formation of the epithelial layer and maintained the crypt length even after DSS treatment. As a potent regenerative agent, the R-spondin 1 protein (Rspo1) has been reported to promote repopulation of the mouse intestinal epithelium, resulting in the amelioration of inflammatory symptoms in not only DSS-induced experimental mice but also IL-10-deficient colitis model mice [51]. Porcine glucagon-like peptide-2 (pGLP-2) is expected to be a potentially effective agent against UC because of its therapeutic effect associated with increased crypt proliferation and the subsequent reduced production of inflammatory cytokines [52,53]. As therapeutic agents, the IJH-SONE68-derived EPSs are expected to have a protective effect on the colonic mucosa, with potent mitogenic activity in crypt cells.

The present study shows that the IJH-SONE68-derived EPS represses the release of IL-8 from the Caco-2 cells stimulated by *S.* Typhimurium or *C. jejuni* (Supplementary Figure S1), which have been reported to enter gut epithelial cells and impair the intestinal barrier [54,55]. The lipopolysaccharide (LPS) produced by these bacteria plays a major role during the invasion. It stimulates toll-like receptor-4 (TLR-4) in the host cell, which leads to inflammatory responses [56,57]. The *Salmonella* flagellin is recognized by TLR-5 as a pathogen-associated molecule, but the *C. jejuni* flagellin is not [58,59]. In bacterial invasion, TLRs interact with at least four types of adapter proteins. One of them is myeloid differentiation factor 88 (MyD88), which can be utilized by all TLRs [60,61]. Makino et al. reported that the EPS from *Lb. delbrueckii* subsp. *bulgaricus* OLL1073R-1 enhances the natural killer (NK) cell activity and interferon (IFN)-γ production in mice [44] through the involvement of MyD88. Moreover, a TLR-4 homolog, RP105, has been reported to recognize not only the LPS but also the phosphorylated EPS from *Lactococcus* (*Lc.*) *lactis* subsp. *cremoris* GCL1176 [62]. Because no bactericidal activities were observed on the IJH-SONE68-derived EPSs, we speculate that the EPSs may play a role in the induction of some cytokines via the same pathway. Consequently, the inhibition of IL-8 release from Caco-2 cells and the UC prevention and improving effect in mice observed in the present study are suggested to be caused by the TLR-4 signaling pathway, as previously predicted in contact dermatitis model mice [15].

The present result suggests that the A-EPS has more potent immune-modulating activity than the neutral one. This characteristic was also observed in a previous study using a contact dermatitis model [15]. In fact, some LAB-derived acidic EPSs have been reported to enhance NK cell activity and induce IFN-γ and IL-1α production [63,64,65]. However, the *Lb. plantarum* No. 14–derived A-EPS and N-EPS, each of which has a different sugar composition and different acidic groups, have shown immunostimulatory activity on Peyer’s patch cells and mesenteric lymph node cells [66]. We have confirmed the hyaluronidase-inhibitory effect on LAB-derived EPSs in previous studies [9,10,11], indicating that the inhibitory activity does not depend on the presence of acidic groups. Therefore, the functional activity of the EPS might be due to its structure or composition rather than its acidic properties.

Due to the disturbed intestinal microbiota, dysbiosis has been found to be one cause of UC. Some studies have demonstrated a benefit of human fecal microbiota transplantation (FMT) to induce remission in active UC [67]. The FMT technique has also been reported to be an effective treatment for other dysbiosis-associated disorders [68,69,70]. Since the exact roles of fecal microbiota have not been fully clarified, patients who have undergone FMT should be monitored carefully. In fact, a systematic review of FMT revealed that adverse effects are not rare, and the estimated mortality rate is estimated to be as high as 3.5% [71]. Furthermore, following the death of a person who underwent FMT in 2019, the United States Food and Drug Administration (FDA) issued a safety alert about the potential for life-threatening infection with multidrug-resistant bacteria after FMT [72].

On the other hand, the therapeutic effect of probiotics has been studied in a number of clinical trials for the treatment of IBD [73,74], showing that the use of probiotics might be recommended for anti-inflammatory therapy in UC patients. In addition to the probiotic cells, the EPS produced by LAB has been reported to confer health benefits such as immune modulation, enhanced NK cell activity, antimicrobial activity, and antitumor activity [44,45,75]. Furthermore, EPS-producing *Lb. delbrueckii* subsp. *bulgaricus* B3 and *Bifidobacterium animalis* A1dOxR can attenuate the oxidative stress in a colitis model and repress inflammatory cytokine production in the differentiated monolayer state of Caco-2 cells, respectively [76,77].

## 5. Conclusions

*Lactobacillus paracasei* IJH-SONE68 produces EPS that may have preventive and improving effects against DSS-induced UC model mice. Although more studies on the structural characterization and structure–function relationship of EPSs are needed, EPSs offer a promising lead for developing not only anti-UC drugs but also those effective against other diseases.

## Figures and Tables

**Table 1:** The criteria for scoring the stool consistency and hematochezia conditions.

Score	Stool Consistency	Hematochezia
0	Normal stools	Normal stools
1	Soft pellets; can be picked up by tweezers	Pale red to red-colored stools; blood spreads only surface on the stools
2	Loose stools; cannot be picked up by tweezers	Red to deep red-colored stools; blood spreads in the stools
3	Diarrhea	Deep red to dark red-colored stools; gross bleeding

**Table 2:** Primers for qRT-PCR used in this study.

Name	Sequence (5′ → 3′)	Target Products
mβ-actin-F	GGGACGACATGGAGAAGA	β-actin
mβ-actin-R	CATACAGGGACAGCACAG	
mCOX-2-F	TCAGTAGGTTTTTGCTGTGAGG	COX-2
mCOX-2-R	GTTCAATGGGCTGGAAGACA	
miNOS-F	GAAGAAAACCCCTTGTGCTG	iNOS
miNOS-R	TTCTGTGCTGTCCCAGTGAG	
mTNF-α-F	CATCTTCTCAAAATTCGAGTGACAA	TNF-α
mTNF-α-R	TGGGAGTAGACAAGGTACAACCC	
mIL-6-F	GAGGATACCACTCCCAACAGACC	IL-6
mIL-6-R	AAGTGCATCATCGTTGTTCATACA	
mMIP2-F	AGTGAACTGCGCTGTCAATG	MIP-2
mMIP2-R	CAAGGCAAACTTTTTGACCG	
mIL-10-F	GGTTGCCAAGCCTTATCGGA	IL-10
mIL-10-R	ACCTGCTCCACTGCCTTGCT	
mIL-12-F	AGGACTTGAAGATGTACCAG	IL-12
mIL-12-R	CTATCTGTGTGAGGAGGG	
mIL-17-F	GCTCCAGAAGGCCCTCAGA	IL-17
mIL-17-R	AGCTTTCCCTCCGCATTGA	
mIL-18-F	CAGGCCTGACATCTTCTGCAA	IL-18
mIL-18-R	TCTGACATGGCAGCCATTGT	

## Data Availability

The data presented in the study are available in article.

## Supplementary Materials

The following are available online at https://www.mdpi.com/article/10.3390/microorganisms9112243/s1, Figure S1: Effects of the IJH-SONE68-derived EPS mixture on the IL-8 levels of a pathogen-stimulated Caco-2 cell line, Figure S2: Effects of LAB-derived EPSs in DSS-induced UC model mice on the changes in disease activity index, Figure S3: Effects of IJH-SONE68-derived EPSs in DSS-induced UC model mice on the mRNA level of (A) COX-2, (B) iNOS, (C) TNF-α, (D) IL-6, (E) IL-12, (F) IL-17, and (G) IL-18 expressed in the colon tissue after 14 days of treatment.
