# Supplementary material for: The Exopolysaccharide Produced by Lactobacillus paracasei IJH-SONE68 Prevents and Ameliorates Inflammatory Responses in DSS–Induced Ulcerative Colitis
Source: Microorganisms. 2021 Oct 28;9(11):2243. doi: 10.3390/microorganisms9112243 (PMC8621803; doi:10.3390/microorganisms9112243)
Supplement: Supplementary file 1 [file microorganisms-09-02243-s001.zip › microorganisms-1416086-supplementary.pdf]

**Supplementary Figure S1.** Effects of the IJH-SONE68-derived EPS mixture on the IL-8 levels of a pathogen-stimulated Caco-2 cell line. At least four replicates of all assays were performed. Abbreviations used in the figure: NC, negative control (without bacterial stimulation); PC, positive control (treated with an *S.* Typhimurium or *C. jejuni* suspension at a multiplicity of infection of 10); EPS, pre-incubated with an EPS mixture prior to pathogen stimulation. The statistical analyses were performed using the Tukey–Kramer multiple comparison test (*, $p < 0.05$; **, $p < 0.01$).

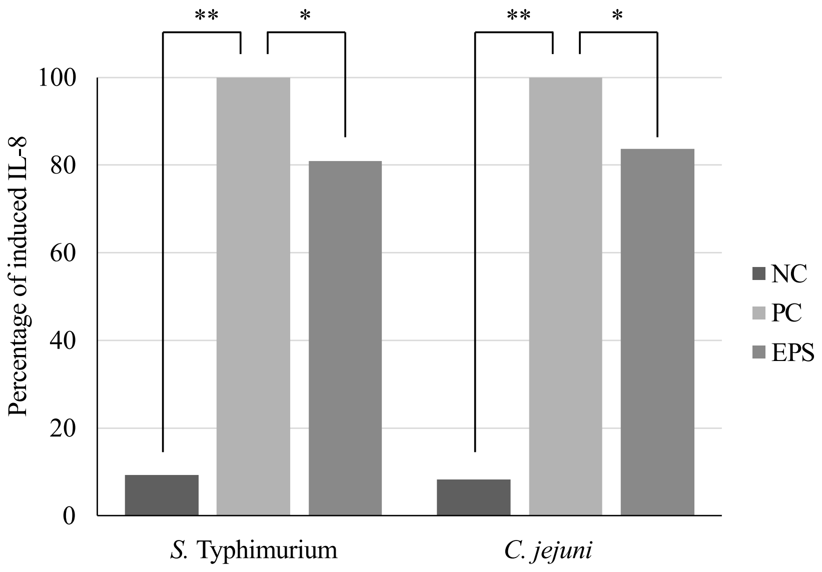
**
*
**
*
100
80
60
40
20
0
Percentage of induced IL-8
NC
PC
EPS
S. Typhimurium
C. jejuni

**Supplementary Figure S2.** Effects of LAB-derived EPSs in DSS-induced UC model mice on the changes in disease activity index. (**A**) Disease activity index at the last day of experimental period using IJH-SONE68- and other strains-derived EPSs. (**B**) Disease activity index at the last day of experimental period using IJH-SONE68-derived N-EPS and A-EPS. Abbreviations used in the figure: NC, negative control group (without UC induction); PC, positive control group (without treatment); N-EPS, UC induction with N-EPS treatment; A-EPS, UC induction with A-EPS treatment. The statistical analyses were performed using the Tukey–Kramer multiple comparison test (*$p < 0.05$, **$p < 0.01$, vs PC). Data are indicated by mean ± S.E. ($n$ = 3–5).

A

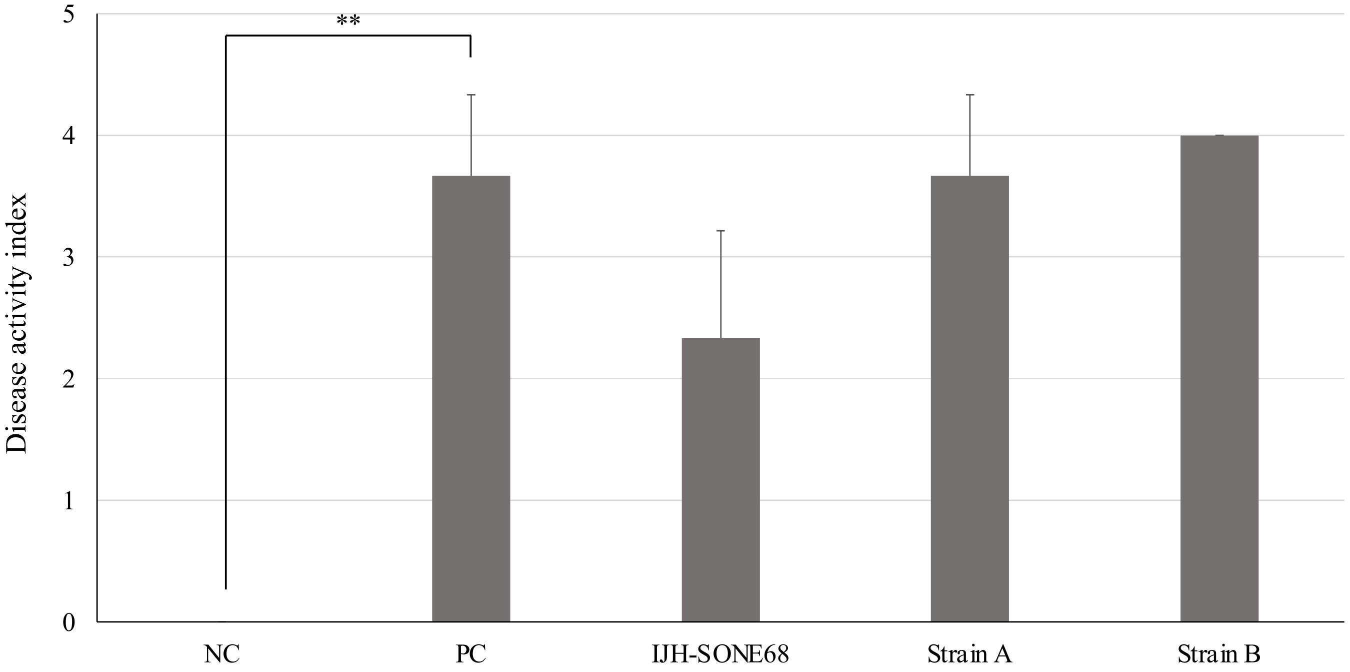
Disease activity index
0
1
2
3
4
5
**
NC
PC
IJH-SONE68
Strain A
Strain B

B

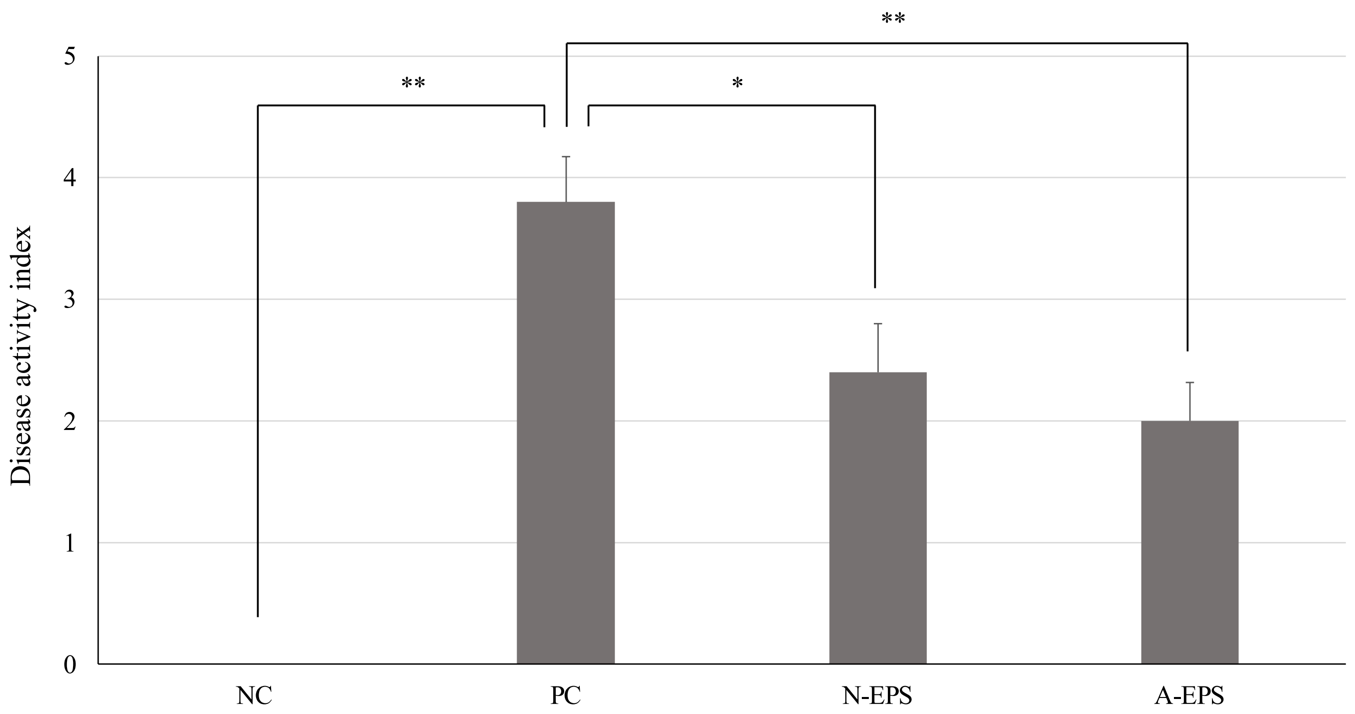

**Supplementary Figure S3.** Effects of IJH-SONE68-derived EPSs in DSS-induced UC model mice on the mRNA level of (**A**) COX-2, (**B**) iNOS, (**C**) TNF-$\alpha$, (**D**) IL-6, (**E**) IL-12, (**F**) IL-17, and (**G**) IL-18 expressed in the colon tissue after 14 days of treatment. Each expression level was normalized to that of the β-actin gene (reference gene) using the $\Delta\Delta C_T$ method. Abbreviations used in the figure: NC, negative control group (without UC induction); PC, positive control group (without treatment); N-EPS, UC induction with N-EPS treatment; A-EPS, UC induction with A-EPS treatment. Data are indicated by mean ± S.E. ($n$ = 4–5).

A

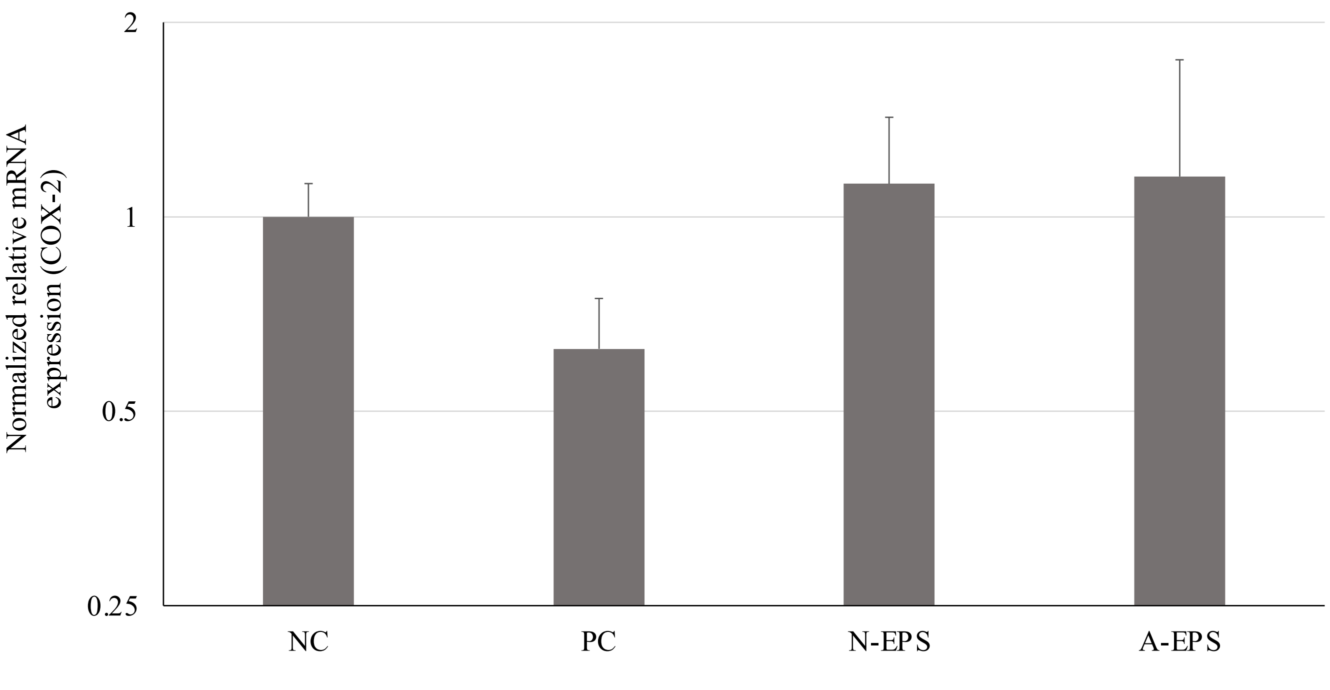
2
1
0.5
0.25
Normalized relative mRNA expression (COX-2)
NC
PC
N-EPS
A-EPS

B

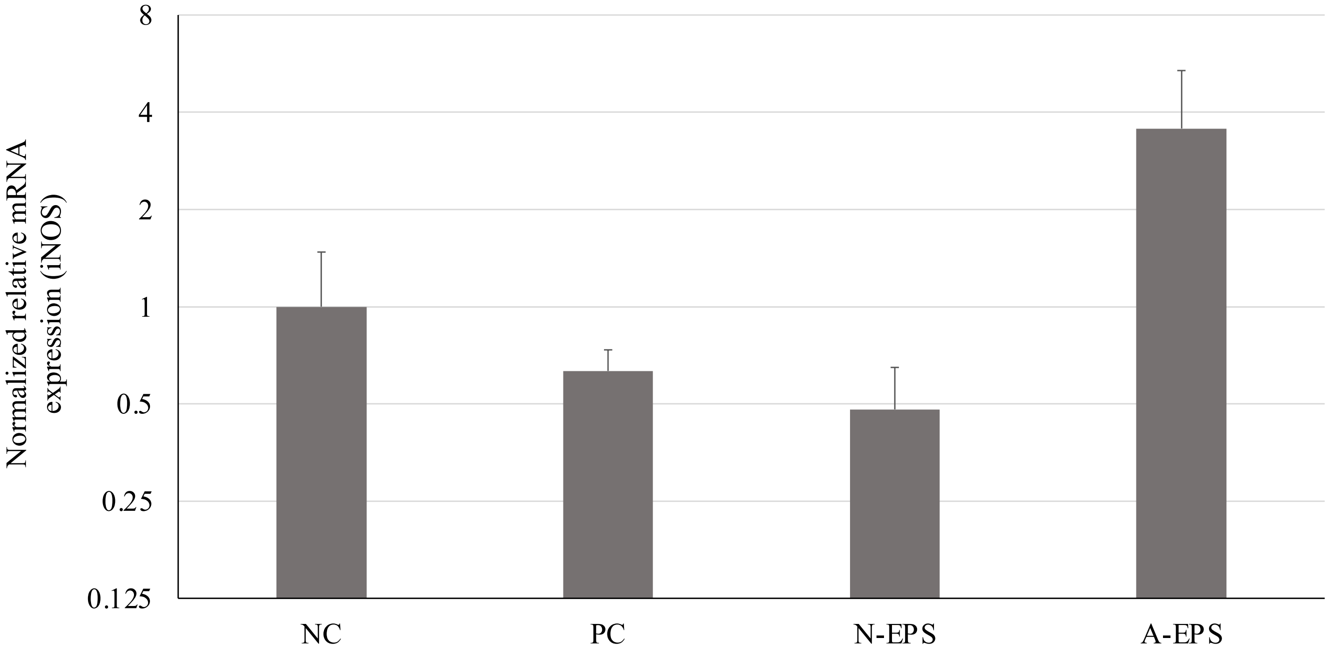

8
4
2
1
0.5
0.25
0.125
Normalized relative mRNA expression (iNOS)
NC
PC
N-EPS
A-EPS

C

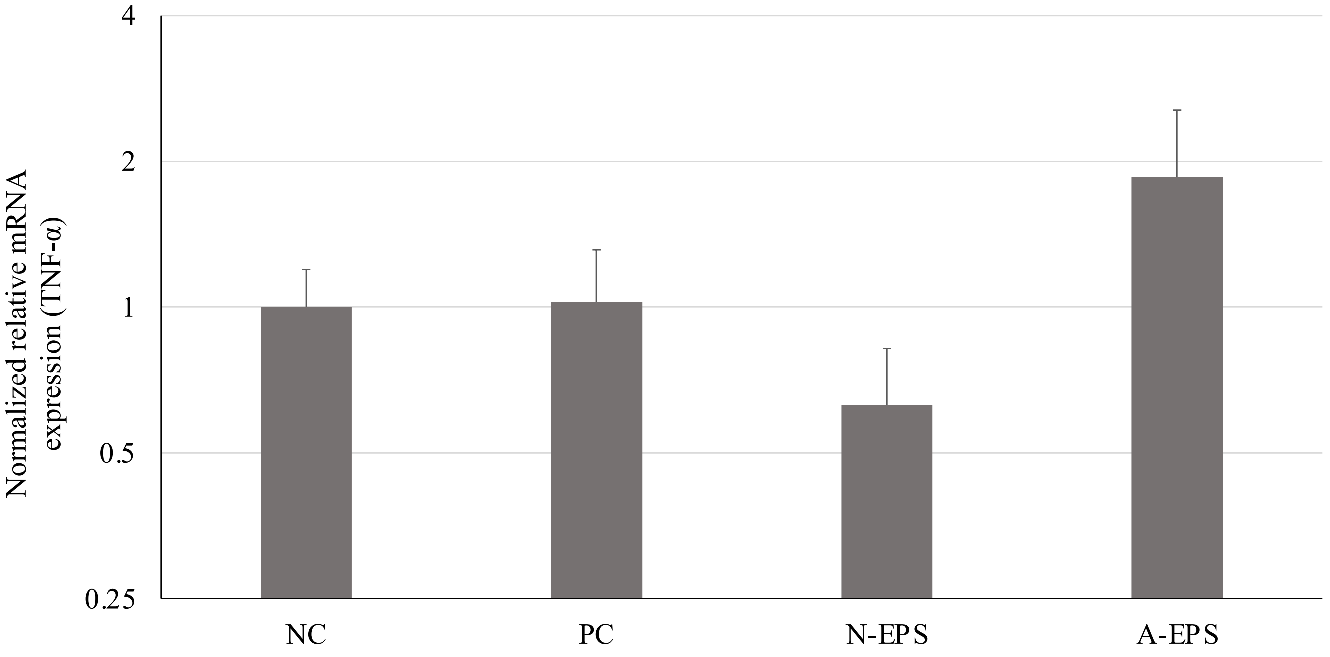
Normalized relative mRNA expression (TNF-α)
4
2
1
0.5
0.25
NC
PC
N-EPS
A-EPS

D

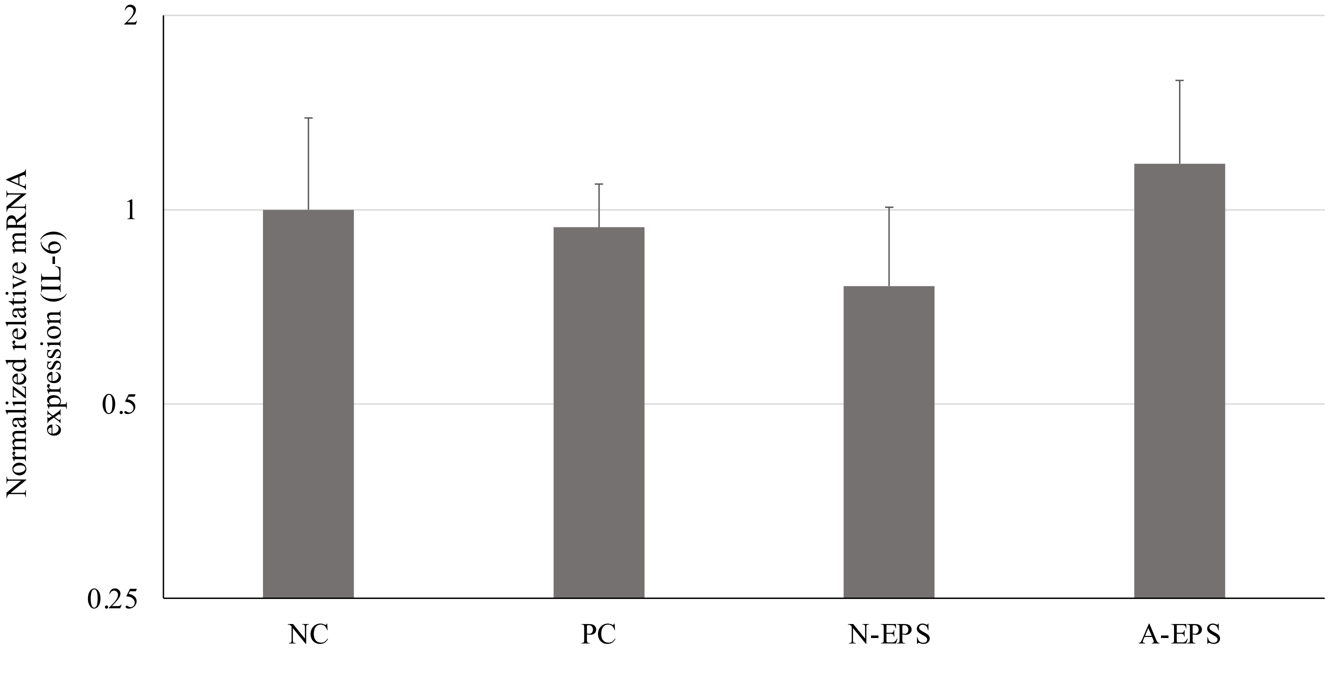
2
1
0.5
0.25
Normalized relative mRNA expression (IL-6)
NC
PC
N-EPS
A-EPS

E

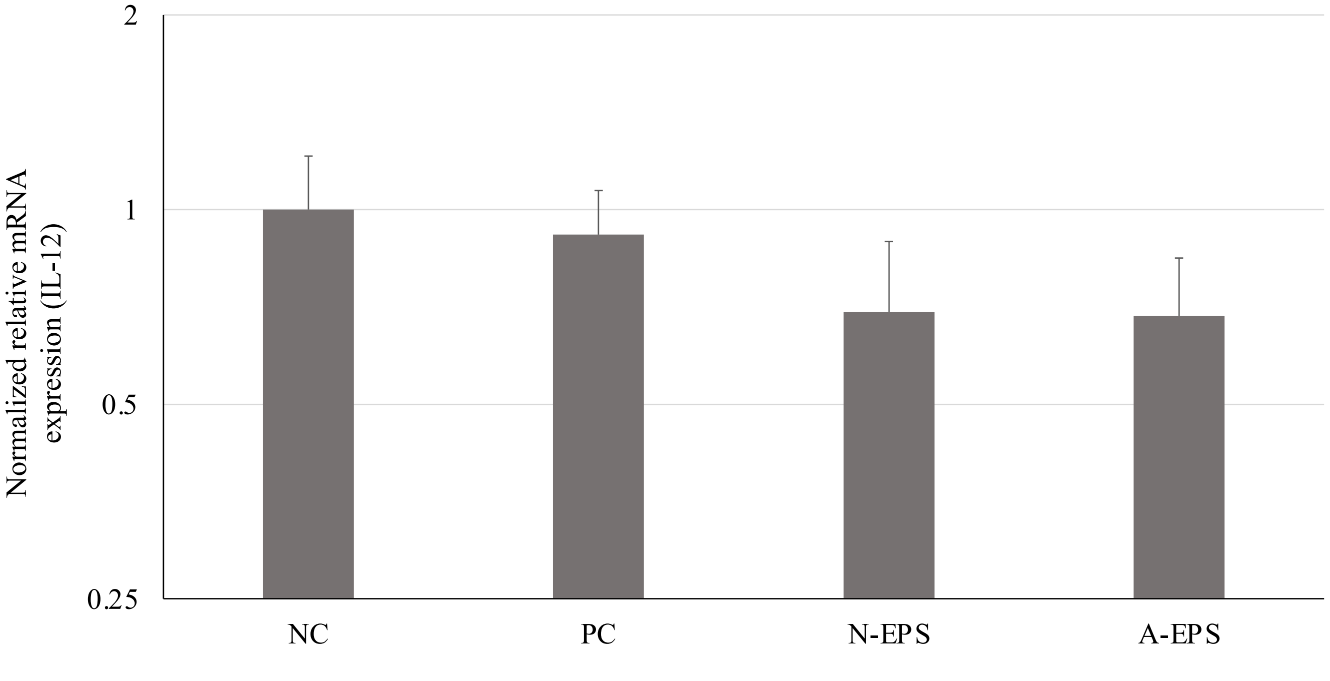
2
1
0.5
0.25
Normalized relative mRNA expression (IL-12)
NC
PC
N-EPS
A-EPS

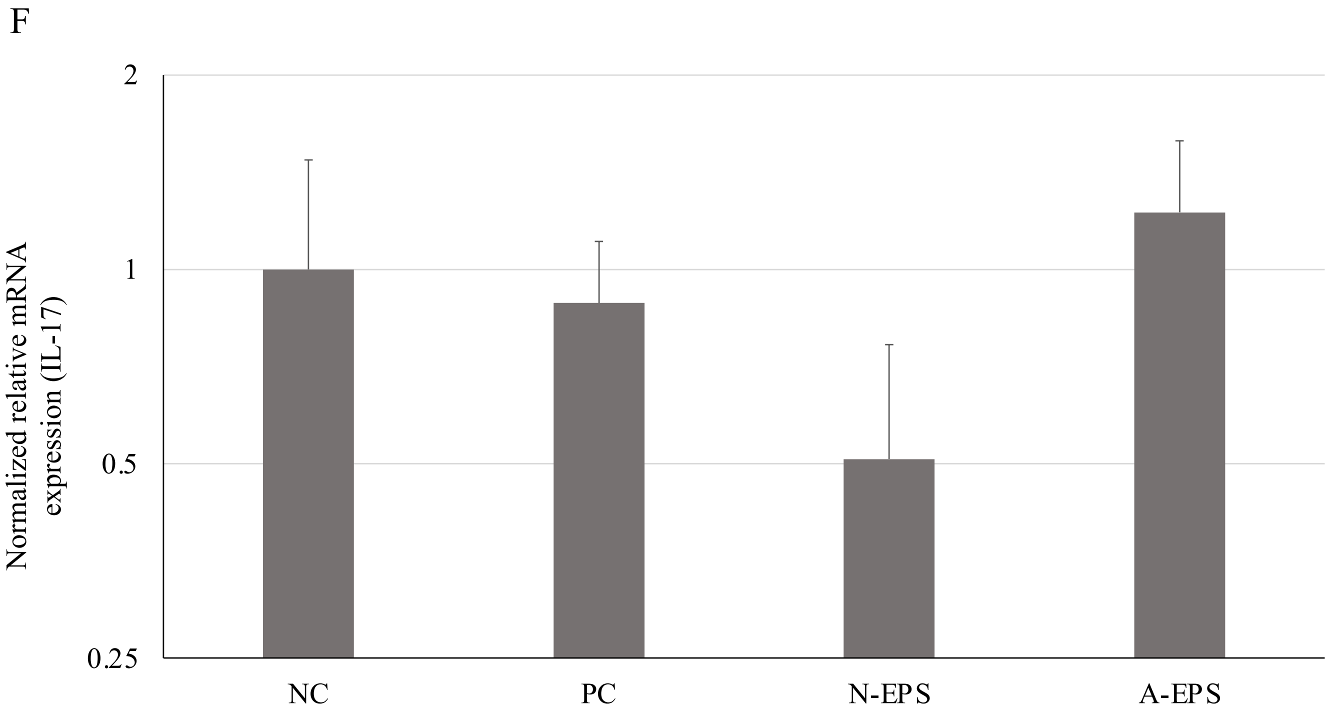
F
2
1
0.5
0.25
Normalized relative mRNA expression (IL-17)
NC
PC
N-EPS
A-EPS

G

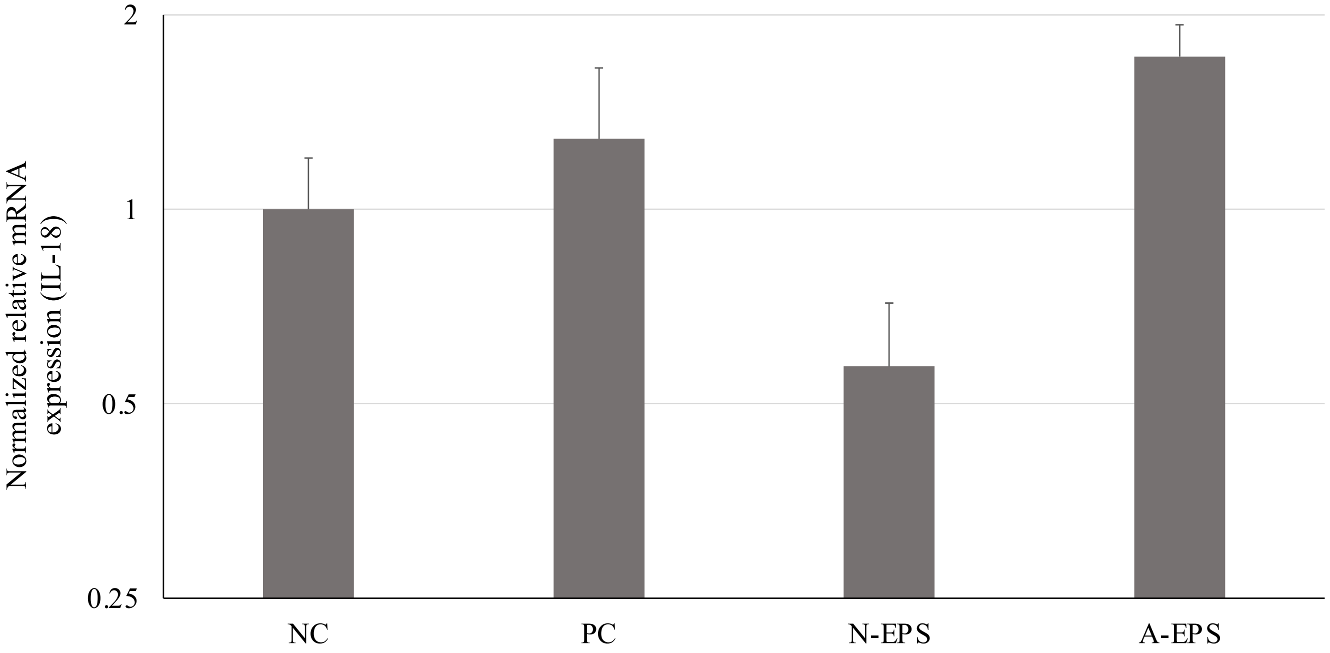
Normalized relative mRNA expression (IL-18)
2
1
0.5
0.25
NC
PC
N-EPS
A-EPS
